# Distinct Roles and Regulations for *Hoxd* Genes in Metanephric Kidney Development

**DOI:** 10.1371/journal.pgen.0030232

**Published:** 2007-12-21

**Authors:** Nicolas Di-Poï, József Zákány, Denis Duboule

**Affiliations:** 1 Department of Zoology and Animal Biology, University of Geneva, Geneva, Swizerland; 2 School of Life Sciences, Ecole Polytechnique Fédérale Lausanne, Lausanne, Switzerland; Medical Research Council Human Genetics Unit, United Kingdom

## Abstract

*Hox* genes encode homeodomain-containing proteins that control embryonic development in multiple contexts. Up to 30 *Hox* genes, distributed among all four clusters, are expressed during mammalian kidney morphogenesis, but functional redundancy between them has made a detailed functional account difficult to achieve. We have investigated the role of the *HoxD* cluster through comparative molecular embryological analysis of a set of mouse strains carrying targeted genomic rearrangements such as deletions, duplications, and inversions. This analysis allowed us to uncover and genetically dissect the complex role of the *HoxD* cluster. Regulation of metanephric mesenchyme-ureteric bud interactions and maintenance of structural integrity of tubular epithelia are differentially controlled by some *Hoxd* genes during renal development, consistent with their specific expression profiles. We also provide evidence for a kidney-specific form of colinearity that underlies the differential expression of two distinct sets of genes located on both sides and overlapping at the *Hoxd9* locus. These insights further our knowledge of the genetic control of kidney morphogenesis and may contribute to understanding certain congenital kidney malformations, including polycystic kidney disease and renal hypoplasia.

## Author Summary

*Hox* genes encode proteins that control embryonic development along the head-to-tail axis and in multiple organs. Here, we show that several members of this gene family are necessary for the normal development of the mammalian kidneys. These genes are clustered in one site on the chromosome and their respective positions within the group determine which component of the kidneys they will contribute to. Using a large collection of engineered mutations in this system, we show that these genes are required both for the growth of the kidneys and for their proper organization, such that mutations in some genes reduce the size of the organs, whereas mutations in others induce polycystic kidneys. Our set of genetic rearrangements also allowed us to localize the position of regulatory sequences, which control the expression of these genes during kidney development.

## Introduction

Mammalian genomes contain 39 genes related to the *Drosophila* homeotic genes, which encode transcription factors necessary for proper development along the major body axis. In the mouse, these *Hox* genes are organized in four separate chromosomal clusters and are expressed along the body axis in a spatio-temporal manner that corresponds to their physical positions along the clusters [1,2]. Because these four clusters were generated by large-scale duplications, *Hox* genes are classified into thirteen groups of paralogy, based on both sequence similarities and respective positions along the clusters. These genes have long been recognized as important regulators of developmental processes, in particular due to their function in specifying regional identities along the anterior to posterior body axis [3].

In the course of tetrapod evolution, *Hox* gene functions were co-opted in a number of developmental processes including the patterning of the limb bud and the formation of several organ systems, where they positively or negatively regulate the expression of genes involved in cell differentiation, adhesion, proliferation and apoptosis [4]. The intricate overlapping expression patterns and structural homology of these gene products underlie many instances of pleiotropy and functional redundancy, phenomena that have substantially complicated their functional analysis. Further untangling of this complex genetic system is expected to yield novel insight into important developmental mechanisms. For example, in the developing mammalian urogenital system, simultaneous expression of about thirty *Hox* genes has been reported, and some traces of colinear regulation became apparent [5–7].

The early molecular events underlying kidney morphogenesis are functionally well described, and are initiated by reciprocal epithelial-mesenchymal signaling interactions between the epithelium of the ureteric bud (UB) and the metanephric mesenchyme (MM) [8]. In mouse, the UB forms during embryonic day 10 (E10) as an epithelial protrusion in the more posterior part of the Wolffian duct. The nearby mesenchyme (future MM) condenses and signals to induce branching morphogenesis of the UB, whereas signals emanating from the UB in turn promote MM survival and activate nephrogenesis. The induced MM expands, becomes a polarized epithelium through the intriguing process of mesenchyme to epithelium transition and forms vesicles that elongate and differentiate to form the nephrons. Molecular genetic analyses in mice have revealed a complex network of regulatory interactions that control kidney organogenesis, involving transcription factors, cell adhesion proteins and secreted molecules [8].

In this context, few studies have addressed the function of *Hox* genes during kidney morphogenesis, except for paralogy group 11; while single mutations in any of the *Hox11* group gene results in normal nephrogenesis, double or triple mutations lead to either absent, or rudimentary kidneys, with a severely reduced number of nephrons [9,10]. Gain of function approaches have suggested a richer variety of HOX function in kidneys, since the over-expression of *Hoxb8* and *Hoxb7* caused kidney hypoplasia and renal duplication, respectively [11,12], whereas the ectopic expression of *Hoxd13* induced renal agenesis [13].

In order to assess the total functional contribution of *Hoxd* genes to kidney development, we performed detailed comparative phenotypic analysis of a set of mouse strains carrying a variety of targeted genomic rearrangements within the *HoxD* cluster. We show that the most striking kidney defect at birth is a failure of UB branching morphogenesis, leading to kidney hypoplasia associated with early postnatal death. This defect correlates with a gain of expression of the posterior *Hoxd12* gene in developing kidneys. Interestingly however, defects reminiscent of polycystic kidney disease (PKD) were also observed, yet exclusively in mutant mice carrying a deletion of more anterior *Hoxd* genes like *Hoxd9* and *Hoxd8*. Finally, the distinct functions of *Hoxd* genes in kidneys correlate well with their specific expression in the MM or UB, respectively. In this context, we use some additional mutant strains to provide evidence for the presence of putative global enhancer and silencer elements, which define and coordinate the expression pattern of *Hoxd* genes in developing kidneys.

## Results

### Multiple Kidney Defects in *HoxD* Cluster Deficiency

**Figure 1:**
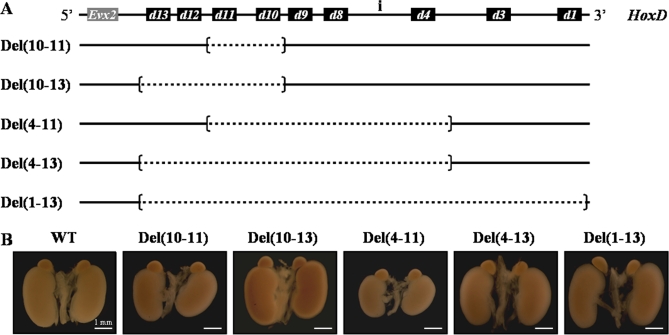
Effects of *HoxD* Cluster Deletions upon Renal Morphology (A) Scheme showing the intact *HoxD* gene cluster (top line) and five strains of mice carrying deletions at the locus which were used to assess the functions of various *Hoxd* genes in kidney development. The intergenic region between *Hoxd4* and *Hoxd8* is referred to as ‘region i'. The neighboring gene *Evx2* is shown as a grey box. Brackets indicate the deletion breakpoints and the deleted fragments are shown in dashed lined. For convenience, the strains are referred to as, e.g., Del(10–11) for a deletion removing from *Hoxd10* to *Hoxd11*, inclusively. Strains are ordered according to the increasing size of the deleted fragment in the *HoxD* cluster. (B) Gross appearance of representative kidneys from control wild type (WT) and *HoxD* mutant newborn mice. The Del(10–11) and Del(4–11) strains exhibit kidney hypoplasia, whereas adrenal glands appeared normal.

As several *Hoxd* genes are expressed during kidney development, multiple modes of functional redundancy or compensatory mechanisms may remain easily hidden in single gene inactivation experiments [7,10]. We thus addressed this question by using more drastic genetic conditions and first compared renal development in a variety of mouse strains carrying targeted deletions of multiple *Hoxd* genes in *cis* (Figure 1A). The shortest of these deletions removed the DNA interval including from *Hoxd10* to *Hoxd11*, and is referred to as Del(10–11), whereas the largest deletion Del(1–13) removed the entire *HoxD* cluster, from *Hoxd1* to *Hoxd13* included (Figure 1A). Other deletions Del(10–13), Del(4–13) and Del(4–11) removed the corresponding intermediate sets of *Hoxd* genes. Importantly, while both Del(10–11) and Del(10–13) deletions removed *Hoxd* genes expressed in the MM only, other deficiencies involved heterogenous sets expressed either in the epithelium, or in the mesenchyme [7].

**Figure 2:**
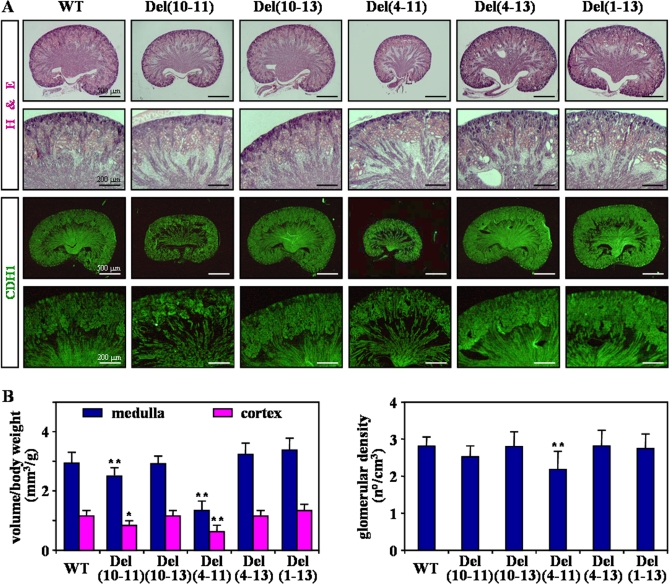
Histology of Neonatal *HoxD* Mutant Kidneys (A) Sagittal sections of newborn kidneys (strains are indicated on the top) were stained with hematoxylin and eosin (H&E, top). At high magnifications, tubular dilatations and large cysts were visible in both the cortical and medullary region of Del(4–11), Del(4–13) and Del(1–13) strains. In the bottom panels, newborn kidneys were processed for the detection of CDH1 in order to identify the UB and its branches. (B) The volume (left) and glomerular density (right) of *HoxD* mutant newborn kidneys were compared with WT control littermates. The volume was separated into distinct cortical (cortex) and medullary (medulla) zones and was expressed as a ratio to body weight (mm^3^/g). The glomerular density represents the number of glomeruli per total volume of kidney (n°/cm^3^). Independent Student's *t* test (*HoxD* deletions versus WT): *, P < 0.05; **, P < 0.01.

While homozygous pups derived from all mutant strains were obtained at birth following a Mendelian proportion, all homozygous Del(4–11) newborns (100%) and between 40 to 50% of the Del(4–13) and Del(1–13) homozygous pups died shortly after birth. When compared to wild-type (WT) littermates at birth, Del(10–13), Del(4–13) or Del(1–13) homozygous mutants displayed grossly normal kidneys, whereas Del(10–11) and Del(4–11) animals showed slight or strong reduction in the size of their kidneys, respectively (Figure 1B). As expected from such severe kidney hypoplasia, histological analysis of Del(4–11) homozygous kidneys revealed a strong reduction of UB branching reflected in a reduced number of collecting tubules in the medulla region, when compared to control and other mutant strains (Figure 2A, top). In addition, UB branching was slightly decreased in Del(10–11) kidneys. Because the severe hypoplasia found in Del(4–11) kidneys was absent from both Del(4–13) and Del(1–13) homozygous mice, we concluded that a mis-regulation of the remaining *Hoxd12* and/or *Hoxd13* genes was likely the cause of this postnatal lethality.

The abnormal reduction in the number of UB branches in Del(10–11) and Del(4–11) newborn kidneys was further documented by using immuno-histochemical detection of CADHERIN type 1 (CDH1), a marker specific for developing UB and epithelial tubules (Figure 2A, bottom). As appropriate branching of the UB is essential to set the number of nephrons, we next determined the volume and glomerular density of WT and mutant kidneys by quantitative morphometry. In both Del(10–11) and Del(4–11) mice, cortical and medullar volumes were reduced when compared to other strains (Figure 2B, left). Furthermore, the density of glomeruli was significantly reduced in Del(4–11) kidneys (Figure 2B, right), further confirming a role for *Hoxd* genes in the control of UB growth.

**Figure 3:**
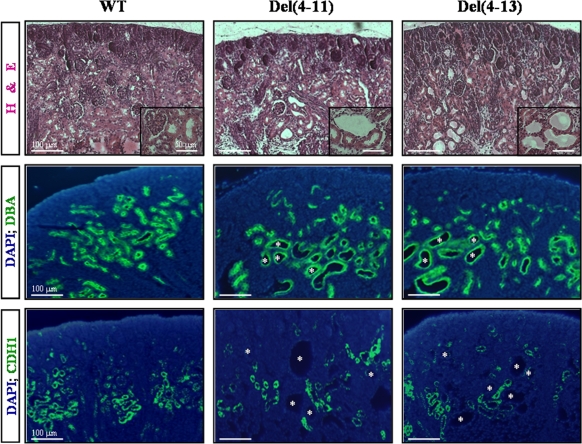
Development of Cystic Kidneys H&E stained-sections illustrate microcystic changes in a subset of tubules in Del(4–11) and Del(4–13) kidneys (top). Cryosections of WT, Del(4–11) and Del(4–13) newborn kidneys were either stained with Dolichos biflorus agglutinin (DBA, middle panels), a specific marker of epithelial tubules, or processed for the detection of CDH1 by immuno-fluorescence (CDH1, bottom panels). Cell nuclei were counterstained with DAPI (DAPI, blue). Renal tubules are shown at higher magnification in the insets.

Histological examination also revealed the presence of tubular dilatations in both the medulla and the cortex of Del(4–11), Del(4–13) and Del(1–13) homozygous newborn kidneys (Figure 2A, top). These defects were observed in heterozygous mutants too, although clearly less frequently. Higher magnifications revealed microcystic changes in these tubules, characterized by abnormally thin epithelial cells and widened lumina, resembling renal cysts observed in PKD (Figure 3, top). Most cysts originated from the UB lineage, as shown by staining with the collecting-duct specific lectin Dolichos bifluoris agglutinin (DBA; Figure 3, middle). In addition, CDH1 immuno-staining, an indicator of cystic kidneys in mice [14], was lost in the plasma membrane of cystic tubules from both Del(4–11) and Del(4–13) homozygous kidneys, suggesting a defect in epithelial cell polarity (Figure 3, bottom). Finally, escaper Del(1–13) homozygous mice, surviving birth for at least one month, developed different degrees of well-visible unilateral or bilateral polycystic kidneys (Figure S1).

Altogether, the abnormal kidney architecture observed in the different *HoxD* deletions suggested important roles for these genes during renal morphogenesis. On the one hand, the hypoplastic phenotype found in both Del(10–11) and Del(4–11) mice involved a mis-regulation of the remaining posterior *Hoxd12* and *Hoxd13* genes, reminiscent of gains of expression as previously seen during the development of the limbs [15]. On the other hand, the microcystic defects were likely induced by the loss of either *Hoxd4*, *Hoxd8* or *Hoxd9*, or combinations thereof, as suggested by their occurrence in Del(1–13), Del(4–13) and Del(4–11), but not in Del(10–11) or Del(10–13) homozygous animals.

### Aberrant Cell Death in *HoxD* Mutant Polycystic Kidneys

**Figure 4:**
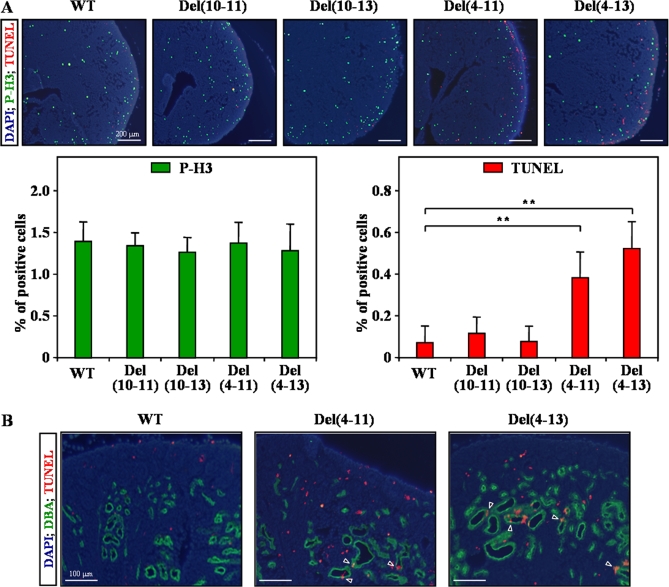
Cell Death in Del(4–11) and Del(4–13) Mutant Kidneys (A) Sections of WT, Del(10–11), Del(10–13), Del(4–11) and Del(4–13) newborn kidneys, double stained for phosphorylated histone H3 (P-H3 mitotic marker, green) and TUNEL (apoptotic marker, red). Cell nuclei were counterstained with DAPI (DAPI, blue). Graphs show the percentage of P-H3- (left) and TUNEL-positive cells (right) among the total population of renal cells. Independent Student's *t* test: **, P < 0.01. (B) Double staining for DBA (green) and TUNEL (red) was performed in order to identify the renal origin of apoptotic cells in the Del(4–11) and the Del(4–13) strains. Apoptotic cells are scored in epithelial microcysts (arrowheads) and in the renal interstitium of mutant kidneys.

Kidney morphogenesis depends upon a delicate balance between cell proliferation and apoptosis. We thus assessed whether the growth defects observed in our *HoxD* deleted mice could derive from modifications in the rates of either one of these processes. Immuno-detection of phosphorylated histone H3 (P-H3), a nuclear protein present in mitotic cells only, showed distribution of proliferating cells in the different compartments of developing kidneys at birth, with relatively higher levels in the cortex (Figure 4A, top). Quantitative analysis of P-H3-positive cells revealed no significant difference between WT kidneys and age-matched Del(10–11), Del(10–13), Del(4–11) or Del(4–13) mutants (Figure 4A, bottom left).

In control newborn kidneys, TUNEL-positive, apoptotic cells were barely detected, either in the nephrogenic zone, or in the medullary papilla (Figure 4A, top). In contrast, apoptosis was increased five to six fold in both Del(4–11) and Del(4–13) kidneys (Figure 4A, bottom right). In these cases, apoptotic cells were particularly numerous in the cortical interstitium as well as in the microcystic tubules previously identified in these two *HoxD* deletions (Figure 4B). No significant changes were observed either in Del(10–11), or in Del(10–13) mutants (Figure 4A), suggesting a critical involvement of the *Hoxd4*-*Hoxd9* genomic interval. Therefore, the precise balance between proliferation and apoptosis was clearly disturbed in both Del(4–11) and Del(4–13) cystic kidneys, with a robust increase in tubular epithelial cell apoptosis, similar to what happens in human and animal models of PKD [16].

### Effects of the Deletions upon Neighboring *Hoxd* Genes

**Figure 5:**
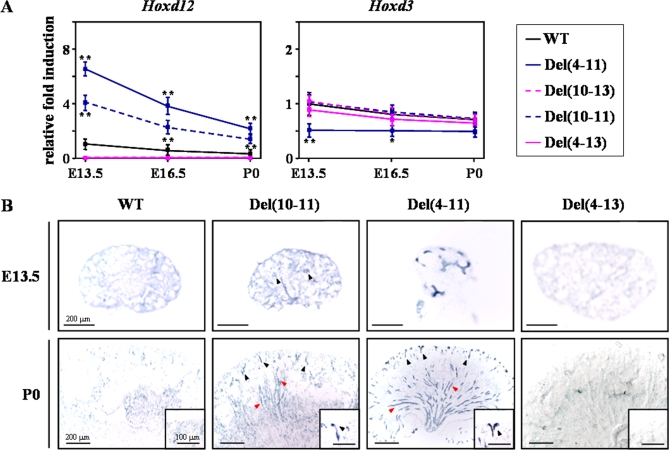
Expression of *Hoxd* Genes Flanking the Deletions (A) qPCR analysis was carried out at embryonic or postnatal days (from E13.5 to P0), using cDNAs generated from control (WT) and *HoxD* mutant kidney mRNAs. Values represent the mean of three independent experiments, after normalization to the WT at day E13.5. Independent Student's *t* test (*HoxD* deletions versus WT): *, P < 0.05; **, P < 0.01. (B) The detection of *Hoxd12* was performed in WT, Del(10–11), Del(4–11) and Del(4–13) kidneys at E.13.5 and P0, by ISH. The Del(4–13) allele was used as a negative control for the specificity of the riboprobe. Del(10–11) and Del(4–11) kidneys showed *Hoxd12* mRNA in some UB branches (red arrowheads) as well as UB tips (black arrowheads and insets).

To explore the molecular mechanisms underlying the UB branching defects, we analyzed the expression pattern of those *Hoxd* genes flanking the internal Del(10–11) and Del(4–11) deletions, using both quantitative RT-PCR (qPCR) and *in situ* hybridization (ISH), to detect potential deletion-induced mis-regulations [15]. By using qPCR and kidneys from embryonic day E13.5 fetuses to postnatal day 0 (P0), we observed a strong gain of expression of *Hoxd12* in Del(4–11) kidneys, and a reproducible increase was also noticed in Del(10–11) mice (Figure 5A). Del(10–13) and Del(4–13) kidneys were used as negative controls for the specificity of *Hoxd12* gene amplification. No (or only very slight) difference was observed in the expression levels of *Hoxd3* under the same circumstances (Figure 5A).

We next analyzed the distribution of *Hoxd12* mRNA by ISH in WT, Del(10–11) and Del(4–11) kidneys at E15.5 and P0, using Del(4–13) as a negative control (Figure 5B). In WT kidneys, *Hoxd12* was expressed rather diffusely within the MM at E13.5, whereas it was detected mainly in the central MM surrounding the main trunk of the UB at birth, with lower expression in MM cells at the periphery of the kidney. In addition to the expression in the MM, Del(10–11) kidneys also showed *Hoxd12* mRNA in some UB branches as well as UB tips, at the junction with the distal segments of the S-shaped body (Figure 5B, bottom). Finally, *Hoxd12* was strongly mis-expressed in the UB branches and tips of Del(4–11) kidneys at both E13.5 and P0. *Hoxd13* expression was undetectable at all these developmental stages, both in the various mutants as well as in WT controls ([7] and data not shown).

These results demonstrated that, as a result of the intervening deletions, the *Hoxd12* transcription unit became progressively mis-expressed and shifted its expression from MM to UB, presumably due to its closer proximity to the more anterior (3') part of the cluster. Importantly, these changes in the quantitative level and tissue distribution of *Hoxd12* expression correlated well with the UB branching defects observed in both Del(10–11) and Del(4–11) mutant kidneys.

### *Hoxd* Regulation of Epithelial-Specific Genes in Developing Kidneys

We investigated the expression of various genes known as regulators of kidney development, in order to identify potential genetic targets of *Hoxd* gene products. We started with the hypoplasia phenotype, common to both Del(10–11) and Del(4–11) mutant mice. The hypoplasia observed in Del(4–11) mice was similar to the effect of *Hoxa11*/*Hoxd11* double loss of function [9], suggesting that the up-regulation of *Hoxd12* expression, in this allele (Figure 5), negatively affects the function of group 11 proteins. A similar mechanism has been proposed for explaining complete kidney agenesis or severe hypoplasia in the TgH[d9/lac] transgenic configuration, wherein the insertion of a transgene near *Hoxd13* up-regulated this latter gene in developing metanephric kidneys [13].

To investigate this hypothesis, we analyzed by qPCR the expression of several genes reported to be strongly mis-regulated in *Hoxa11*/*Hoxd11* double mutant kidneys [17]. However, both Del(4–11) and Del(4–13) homozygous kidneys exhibited normal expression of all genes assayed in this respect (Figure S2). In contrast, similar analyses carried out on the TgH[d9/lac] transgenic mice revealed a significant increase of both *Gata6* and *Tgfβ2* expression, two genes described as target of *Hoxa11*/*Hoxd11* [17], together with a massive gain of expression of *Hoxd13* in the MM and subsequent kidney hypoplasia (Figure S3).

**Figure 6:**
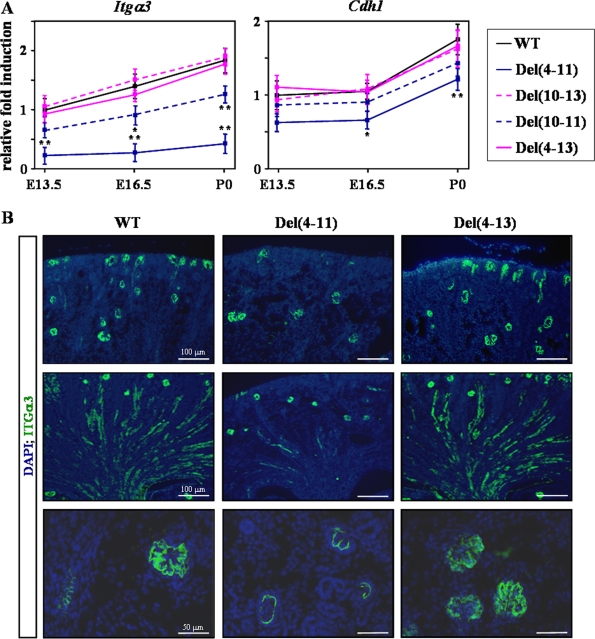
Expression of Adhesion Molecules in *HoxD* Mutants (A) Expression of *Itgα3* and *Cdh1* was assessed by qPCR from E13.5 to P0. Independent Student's *t* test (*HoxD* deletions versus WT): *, P < 0.05; **, P < 0.01. (B) In newborn kidneys, the ITGα3 protein was abundant in the UB and in glomeruli, as revealed by immuno-fluorescence (green). However, ITGα3 immuno-staining was largely reduced in UB tips (top) and branches (middle) of Del(4–11) mutant when compared to WT and Del(4–13) kidneys. In addition, the basement membrane structure of Del(4–11) mutant glomeruli was disorganized (bottom). Cell nuclei were counterstained with DAPI (blue).

Because the gain of *Hoxd13* expression in the MM of TgH[d9/lac] transgenic kidneys involved modifications in MM-specific genes (*Gata6* and *Tgfβ2)*, we investigated whether the gain of posterior *Hoxd* gene expression in the UB of Del(4–11) mice could affect the expression of UB-specific genes, instead of the previously tested MM candidates. Little (if any) difference was scored amongst the various *HoxD* strains, upon analyses of the expression levels of a battery of genes known to regulate UB branching (Figure 6A and S4A). Interestingly however, a severe reduction of embryonic and postnatal *integrin-α3* (*Itgα3*) gene expression was observed in Del(4–11) hypoplastic kidneys, as compared to their WT and Del(4–13) counterparts (Figure 6A). A slight down-regulation of *Itgα3* was already present in the Del(10–11) kidney, whereas a normal expression level was found in the TgH[d9/lac] transgenic strain (data not shown).

The *Itgα3* gene was of particular interest in this context, since its functional inactivation leads to mice with similar defects in branching morphogenesis and microcystic phenotypes than those observed in our Del(4–11) mutants [18]. Altered expression of *Itgα3* in Del(4–11) kidneys was further confirmed at the protein level by using immuno-fluorescence. ITGα3 is present in both the UB and glomerular podocytes, along the glomerular basement membrane (GBM) of WT kidneys (Figure 6B and reference [19]). Strikingly, ITGα3 immuno-staining was virtually lost in Del(4–11) mutant UB branches and tips, when compared to WT and Del(4–13) controls (Figure 6B, top and middle). In addition, the GBM was disorganized in a number of Del(4–11) mutant kidneys, as shown by reduced capillary branching (Figure 6B, bottom). These glomerular anomalies are consistent with an essential role of ITGα3 in the maturation of the GBM [20], and may result in the loss of normal glomerular filtration barrier in Del(4–11) kidneys.

### *Hoxd* Control of Apoptosis in Renal Tubules

**Figure 7:**
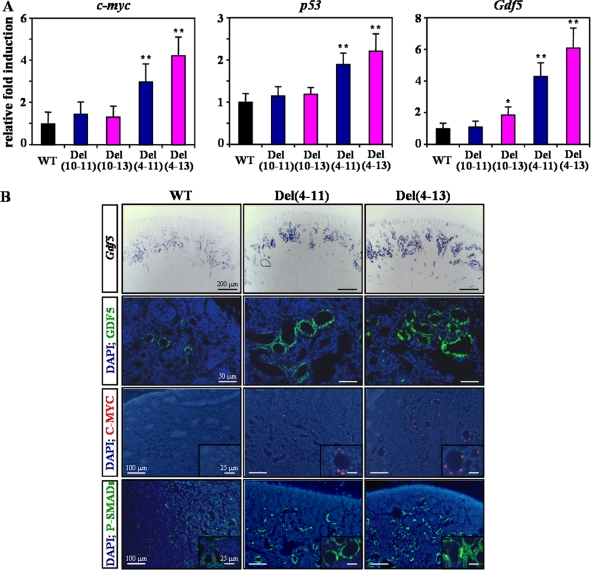
Expression of Apoptotic-Related Genes in *HoxD* Mutant Kidneys (A) qPCR measurements of the indicated genes were performed in newborn kidneys from WT, Del(10–11), Del(10–13), Del(4–11) and Del(4–13) strains. Values represent the mean of three independent experiments, after normalization to the WT. Independent Student's *t* test (*HoxD* deletions versus WT): *, P < 0.05; **, P < 0.01. (B) An increased expression of *Gdf5* in the epithelial tubules of Del(4–11) and Del(4–13) newborn kidneys was observed both at the mRNA and protein levels (top). C-MYC and phospho-SMADs (bottom) were also up-regulated in renal tubules. Tubules are shown at higher magnification in the insets. Cell nuclei were counterstained with DAPI (blue).

We then focused on the polycystic kidney phenotype, common to both Del(4–11) and Del(4–13) mutant mice. Because these cystic kidneys displayed increased apoptotic activity (Figure 4), we used qPCR to determine the expression levels of those apoptotic genes known to trigger a cystogenic pathway in mouse dysplastic kidneys (Figure 7A and S4B) [21]. The RNA content of *c-myc* and *p53*, two important pro-apoptotic gene markers of PKD [16], were both increased in Del(4–11) and Del(4–13) kidneys, when compared to WT controls (Figure 7A). Expectedly, C-MYC was detected in a nuclear pattern in epithelial cystic tubules of both Del(4–11) and Del(4–13) kidneys (Figure 7B). In addition, we scored a strong up-regulation of *growth and differentiation factor-5* (*Gdf5*) in these two mutant kidneys, coincident with the observed cystic dilatations of epithelial tubules. *Gdf5* mRNA and protein expression levels were elevated in Del(4–11) and Del(4–13) kidneys, in both non-cystic and cystic tubules, as revealed by ISH and immuno-fluorescence, respectively (Figure 7B, top).

Members of the TGFβ super-family trigger cellular responses through the SMAD protein pathway, which transduces the signal from the cell surface to the nucleus to activate specific gene transcription [22]. We thus analyzed the expression of phosphorylated SMAD 1-5-8 proteins (P-SMADs) in cystic kidneys, as a cellular reporter of TGFβ pathway activation. Although P-SMADs were expressed at low levels, in a cytoplasmic pattern in epithelial tubules from control tissue, they were robustly up-regulated in both the cytoplasms and nuclei of microcystic tubules from Del(4–11) and Del(4–13) kidneys (Figure 7B), suggesting an increased activity of the BMP/GDF pathways in these tubules.

### *Hoxd8* and *Hoxd9* Regulate Epithelial Microcyst Formation

The polycystic kidney phenotype was observed only in those *HoxD* cluster deletions which remove both anterior and posterior *Hoxd* genes, such as Del(4–11), Del(4–13) and Del(1–13). In contrast, this phenotype never developed in those strains where only posterior genes had been affected, such as Del(10–11) and Del(10–13), suggesting that this alteration was caused by (a) gene(s) lying within the *Hoxd9* to *Hoxd4* interval (Figure 2).

**Figure 8:**
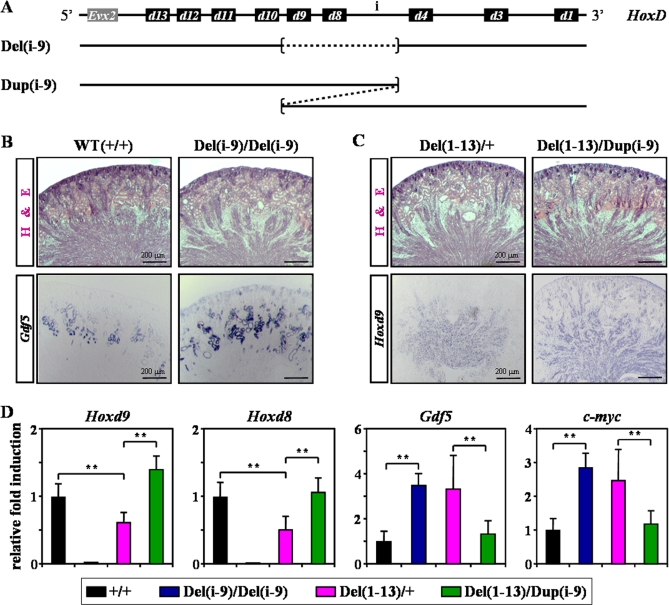
Deletion of Both *Hoxd8* and *Hoxd9* Leads to Epithelial Microcyst Formation (A) Scheme showing the intact *HoxD* cluster (top) and two newly produced mouse strains used to characterize *Hoxd* genes responsible for microcyst formation. The strains are referred to as Del(i-9) or Dup(i-9) for a deletion or a duplication, respectively, and they span from ‘region i' to *Hoxd9*, inclusively (dashed lined). (B, C) Kidney cryosections of WT (+/+), Del(i-9) homozygous (Del(i-9)/Del(i-9), Del(1–13) heterozygous (Del(1–13)/+) and Del(1–13)/Dup(i-9) compound mice were either stained with H&E (top), or processed for the detection of *Gdf5* (left) or *Hoxd9* (right) by ISH. The Del(1–13)/Dup(i-9) compound strain was obtained by breeding Del(1–13) and Dup(i-9) heterozygous mice. (D) qPCR was used to compare the expression levels of *Hoxd9*, *Hoxd8*, *Gdf5* and *c-myc* in newborn kidneys from the mouse strains described before. Values represent the mean of three independent experiments, after normalization to the WT. Independent Student's *t* test: **, P < 0.01.

To further clarify the importance of these genes in the formation of microcysts, we used two novel mutant strains, carrying either a deletion or a duplication of the region located between *Hoxd9* and the intergenic ‘region i' (Del(i-9) and Dup(i-9); Figure 8A), i.e. deleting or duplicating in *cis* both *Hoxd9* and *Hoxd8*. Histological examination of Del(i-9) homozygous kidneys revealed the presence of epithelial microcysts, as well as an increased tubular *Gdf5* expression (Figure 8B). In this allele, the expression of neighboring posterior and anterior *Hoxd* genes was not importantly modified, strongly arguing against any potential global regulatory re-allocations that may have triggered a cystic phenotype (Figure S5 and data not shown). As mentioned earlier, microcystic tubules were also observed in Del(1–13) heterozygous kidneys, with a penetrance of about 50 percent (3 out of 6 mice), consistent with the loss of function explanation involving *Hoxd8* and *Hoxd9*. Interestingly, these renal defects were never observed in six compound mutant mice generated on the same genetic background, which were trans-heterozygous for Del(1–13) and Dup(i-9), suggesting a rescue from the duplicated allele (Figure 8C, top). In these Del(1–13)/Dup(i-9) animals, the two copies of *Hoxd9* and *Hoxd8,* coming from the duplicated DNA segment were likely expressed at similar levels, since expression was about two-fold higher than in Del(1–13) heterozygous kidneys (Figure 8D). However, while *Hoxd9* expression level was similar in the MM, it was clearly higher in the UB, suggesting that the second *Hoxd9* gene copy was mostly expressed in the UB (Figure 8C, bottom). By using whole mount *in situ* hybridization, *Hoxd8* was detected only in the UB of WT and Del(1–13) kidneys, with relatively higher expression in Del(1–13)/Dup(i-9) kidneys (data not shown). Accordingly, the expression of *Gdf5* and *c-myc* also decreased in Del(1–13)/Dup(i-9) kidneys, reaching levels observed in WT conditions (Figure 8D). These results suggest a role for *Hoxd9* and *Hoxd8* (and perhaps for ‘region i') in the regulation of tubular epithelia in postnatal kidneys.

### Looking for Mesenchymal and Ureteric Bud Enhancers

**Figure 9:**
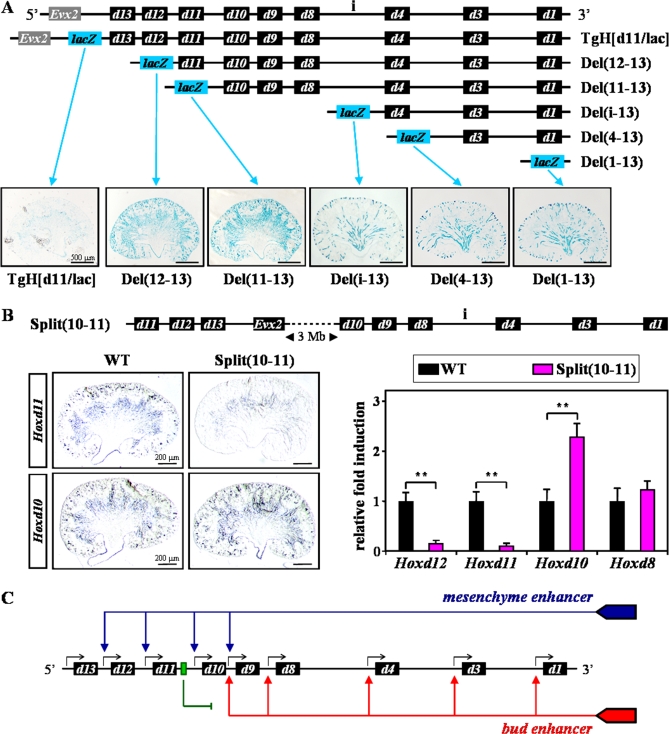
Searching for Putative Mesenchyme and Ureteric Bud Enhancers (A) Schematic representation of a panel of *in vivo HoxD* alleles containing the *Hoxd11/lacZ* fusion reporter gene together with progressive deletions of the cluster. Blue boxes (*lacZ*) indicate the position of the same reporter transgene at the posterior end (5', left) of the intact (TgH[d11/lac]) or deleted (Del) *HoxD* cluster. Representative stainings of newborn kidneys are shown below. (B) Scheme of the inversion-mediated split of the *HoxD* cluster between *Hoxd11* and *Hoxd10* (top; Split(10–11)) [23], separating the two half-clusters by approximately 3 Mb. ISH for *Hoxd11* and *Hoxd10* genes (left), as well as qPCR for *Hoxd12*, *Hoxd11*, *Hoxd10* and *Hoxd8* (right) were carried out in WT and Split(10–11) specimen. Independent Student's *t* test: **, P < 0.01. (C) Model of global *Hoxd* gene regulation during kidney development. The different expression domains are likely controlled by regulatory elements localized outside the *HoxD* complex, at the telomeric (3') side. The bud enhancer may control the expression of anterior genes (from *Hoxd9* to *Hoxd1*) in the UB (red arrows). Posterior *Hoxd* genes are not responsive to this enhancer, because of the presence of a putative silencer or boundary element (green arrow), located between *Hoxd10* and *Hoxd11*. The mesenchyme enhancer may trigger the expression of posterior genes (from *Hoxd12* to *Hoxd9*) in the MM.

In order to determine the influence of *Hoxd* genes' clustered organization on their transcriptional regulation in MM and UB kidney cell types, we used a collection of mouse strains containing the same reporter transgene together with progressively more extended deletions of the *HoxD* cluster (Figure 9A, top). In these strains of mice, a *Hoxd11/lacZ* fusion gene effectively replaced the deleted regions and thus allowed for histochemical detection of β-galactosidase activity in kidneys. In this way, the transcriptional behavior of the same reporter gene could be assessed when located at various positions within the gene cluster. As expected from the absence of *Hoxd13* expression in kidneys, reporter activity was not detected when the fusion gene was positioned at the posterior end of the *HoxD* complex flanked by *Hoxd13* on its 3' side (TgH[d11/lac]; Figure 9A, bottom). However, the proximity of progressively more 3' parts of the *HoxD* cluster induced accompanying progressively more anterior shifts of reporter gene expression along the trunk (data not shown), including a shift from MM to UB. While Del(12–13) and Del(11–13) strains showed strong β-galactosidase activity in the MM, only UB but no MM cells were stained when the deletions brought the transgene closer to the 3' (telomeric) part of the cluster (Figure 9A, bottom). These expression patterns were suggestive of the existence of two distinct enhancer sequences, one controlling expression of ‘posterior' genes in the MM, the other triggering expression of more ‘anterior' *Hoxd* genes in the UB.

Because reporter transgene activity in the UB persisted even when the *HoxD* cluster was entirely deleted, a putative UB enhancer ought to localize outside the cluster itself. On the other hand, reporter gene activity in the mesenchyme was scored in the Del(11–13) strain, whereas absent in Del(i-13) animals, suggesting that a mesenchyme enhancer activity may reside within the cluster, between *Hoxd10* and ‘region i'. However, in mice lacking either halves of this region (i.e. Del(i-9) or Del(10–11)), expression of the remaining posterior *Hoxd* genes in the MM was still clearly detected (Figure 5B and S5). From these observations, we conclude that the absence of reporter activity in the MM of Del(i-13) mice is not due to the deletion of the corresponding enhancer sequence. Consequently, this putative regulatory sequence must lie either in the most anterior part of the cluster (within the *Hoxd4* to *Hoxd1* region) or, like for the UB regulation, outside the cluster itself.

Because enhancers controlling *Hoxd* gene expression in limbs are functionally separated and distributed on either sides of the cluster [23], we further tried to map these putative ‘kidney enhancers'. To this aim, we used mice carrying a large inversion that splits the *HoxD* cluster into two parts, between *Hoxd11* and *Hoxd10* [23], separated by a 3 Mb large DNA fragment (Figure 9B, top). In such mice, the *HoxD* cluster is split into two sub-clusters with independent regulations [23]. In WT newborn kidneys, the expression of both *Hoxd11* and *Hoxd10* was detected in the MM only (Figure 9B, left). Interestingly, after separating the complex, mutant kidneys completely lost expression of both *Hoxd11* and *Hoxd12*, whereas expression of *Hoxd10* in the MM remains unchanged. Quantitative analyses by using qPCR confirmed the abrogation of *Hoxd12* and *Hoxd11* expression in this allele, whereas *Hoxd10* or other UB-specific genes, such as *Hoxd8*, exhibited slightly higher than normal expression levels, respectively (Figure 9B, right). Altogether, these results strongly suggest that the putative global enhancer sequences controlling the expression of *Hoxd* genes in both MM and UB are located either within the most telomeric part of the *HoxD* cluster, or further outside in 3'.

## Discussion

In this paper, we report distinct roles for *Hoxd* genes during kidney development. While the most posterior genes (from *Hoxd13* to *Hoxd11*) are involved in the regulation of metanephric mesenchyme-ureteric bud interactions (this study and [10]), genes located at more ‘anterior' positions, such as *Hoxd9* and *Hoxd8,* are required to maintain the integrity of the renal tubular epithelia. The gain of function of *Hoxd12* was indeed associated with defects in UB branching and glomeruli formation, in parallel with a down-regulation of the cell adhesion molecule ITGα3. On the other hand, the absence of anterior gene functions, including that of *Hoxd9* and *Hoxd8*, lead to the development of polycystic kidneys, likely resulting from the activation of the BMP/GDF apoptotic signaling pathway in epithelial tubules. Finally, this work suggests a model whereby global regulatory sequences, located on the same side of the cluster, define the allocation of particular *Hoxd* genes either to mesenchymal cells, or to epithelial parts of the developing kidney, due to their respective genomic position within the gene cluster.

### Posterior *Hox* Genes Are Important Regulators of Kidney Morphogenesis

A high proportion of *Hox* genes are expressed in both human and mouse kidneys [6,7] and several reports have pointed to an important role for these genes during renal development. Functional studies in mice have illustrated the functional overlap that exists between paralogous genes as well as between genes that are adjacent to each other in the same cluster. While a single mutation in any gene of paralogous group 11 results in normal nephrogenesis, double or triple mutations lead to either absent, or rudimentary kidneys, indicating a critical role for paralogous group genes in UB branching morphogenesis [9,10]. Furthermore, the combined inactivation of *Hoxa10* and *Hoxd10* altered both the position and the size of developing kidneys [24], and some renal size reductions were also scored in *Hoxd9*/*Hoxd10* double mutant mice [25].

Here, we show that TgH[d9/lac] and Del(4–11) mutant mice have a reduced branching morphogenesis. In the former case, we explain this by the strong gain of *Hoxd13* expression in developing kidney mesenchyme, which negatively affects the function of group 11 paralogs, through a process known as ‘posterior prevalence', whereby the product of posterior genes can override the function of more anterior products [26]. Ectopic *Hoxd13* expression led to a failure of the mutant MM to properly induce UB branching, similar to the phenotype of combined group 11 loss of functions. In the latter case, the gain of function involves *Hoxd12* in the UB, which leads to branching inhibition as well as podocyte GBM defects, coherent with the down-regulation of the cell adhesion molecule ITGα3.

Although experimental evidence supports a link between posterior *Hoxd* genes and *Itgα3* in developing kidneys, the demonstration of a direct interaction between these components, so far hampered by the lack of fully specific anti-HOX antibodies, remains to be established. Firstly, *Itgα3* deficient newborn mice exhibit UB branching and GBM defects similar to those reported here, due to impaired epithelial cytoskeleton organization [19]. Secondly, α3 subunit-containing integrins bind with high affinity to isoforms of laminin-10 and −11, which are the predominant integrin ligands in both the UB and mature glomeruli [19]. Interestingly, targeted disruption of these laminins leads to hypoplastic kidneys [18]. Also, accumulating evidence suggests the existence of functional links between these two groups of genes in a variety of tissues and contexts [27]. In mouse kidney cell lines, for instance, *Itgα8* and *Iap* are targets of *Hoxa11* [28], whereas the *ITGα3* gene is regulated by HOXD3 and HOXD10 in some human cancers [29,30]. Finally, *Hox* genes may regulate the progression of human kidney diseases, including glomerulonephritis, diabetic nephropathy and PKD, where integrins have been reported to play an important role [19].

### *Hoxd* Genes in Polycystic Kidney Disease

PKD, one of the most frequent human genetic disorders, constitutes a major cause of end stage renal failure worldwide [21]. In human and rodent models of PKD, pathological features include increased apoptosis, as well as dedifferentiation, adhesion and ciliary abnormalities of epithelial cells [16]. Cystic kidneys were previously observed in a fraction of *Hoxd11* null mice containing a chimeric *Hoxa11* allele with the *Hoxa4* homeobox [31]. In this work, we show that the combined deletion of *Hox* gene functions (*Hoxd9* and *Hoxd8*) leads to the development of polycystic kidneys. The comparative analysis of renal development and gene expression in the various *HoxD* deleted strains also revealed a significant increase of the cystic phenotype in Del(4–13) and Del(1–13) mice, as compared to Del(4–11) and Del(i-9), suggesting some participation of *Hoxd12* in the regulation of the mesenchymal-epithelial transition in developing tubules. In previous studies, this loss of function phenotype may have been compensated for by functional redundancy, as illustrated either for the group 11 *Hox* genes [10], or between non paralogous genes expressed in similar kidney compartments [7].

Of note, mice lacking either all group 9 or all group 8 genes die soon after birth for unknown reasons [32,33], which may illustrate a protective role of *Hoxd9* and *Hoxd8* against the development of PKD. Also, loss of these genes was reported to have occurred in different human cancers affecting kidneys, the thyroid and breast tissues [34,35], even though a direct causal relationship was not established. In addition, a detailed analysis of the set of genes mis-regulated in a murine model of PKD revealed no difference (or a slight decrease) for *Hoxd9* and *Hoxd8* expression, whereas the neighboring *Hoxd10*, *Hoxd4* and *Hoxd3* were all increased [36]. These observations support the hypothesis that *Hoxd9* and *Hoxd8* genes might be necessary to maintain a given differentiated phenotype in epithelial cells.

In mice, PKD can be induced by mutations in a variety of genes, including several members of the BMP/GDF family of proteins such as BMP4 and BMP7 [37]. Here, we report the up-regulation of another member of the family, GDF5, in *Hoxd* mutant polycystic kidneys. Like BMPs, GDF proteins trigger cellular responses through their binding to type-I receptors (preferentially ALK3 and ALK6) and subsequent activation of the SMAD pathway (Smad 1-5-8), which relays the signal from the cell surface to the nucleus to activate specific gene transcription [22]. Interestingly, both ALK3 and ALK6 receptors are highly expressed in developing kidneys [37] and transgenic mice over-expressing a constitutively active form of ALK3 in the UB lineage develop PKD [38]. In our different *HoxD* deletions that induce PKD, down-regulation of CDH1 and up-regulation of P-SMADs and C-MYC expression levels were observed, similar to what was scored in cystic tubules from ALK3 transgenic mice. We consider this as an indication that the activation of the BMP/GDF pathway, in our mutants, may be triggered by this receptor. In this respect, it is noteworthy that the primary function of ALK3 seems to be associated with apoptosis, at least in developing limbs [39].

### Model of *Hoxd* Gene Regulation during Kidney Development

The distinct functions observed at the genetic level for some ‘posterior' and ‘anterior' *Hoxd* genes in kidneys correlate well with their specific expression in either the MM or the UB, respectively ([7] and this work). The data obtained using our collection of mutant strains suggest that the distribution of *Hoxd* genes between these two renal compartments is under the control of global enhancers, resembling in this respect the regulation of the *HoxD* cluster in the developing limbs and intestinal hernia [23]. In developing kidneys, a putative mesenchyme enhancer may control the expression of *Hoxd12* to *Hoxd9*, whereas a ureteric bud enhancer may control gene expression from *Hoxd9* to *Hoxd1*, *Hoxd9* being thus the only gene responsive to both enhancers (Figure 9C).

Because these expression specificities concern two different cell types, it is likely that the regulation of *Hoxd* gene expression in kidneys involves two distinct enhancer sequences. While the data shown here suggest that both of these sequences unexpectedly reside at the telomeric end (or side) of the cluster, their precise nature and location will have to await a more extensive transgenic approach of the suspected genomic region. As it stands, we cannot rule out the possibility that a single global enhancer, controlling expression in both MM and UB, may be at work, involving various cell-type or tissue-specific co-factors. Regardless of the number of enhancers involved, the underlying mechanism(s) must be complex to account for the quasi exclusion of the two expression specificities, with the exception of *Hoxd9.* The analysis of yet a few more genetic configurations will shed some light on this issue, for example whether part of these expression differences are due to the presence of silencer or boundary elements, as reported in similar instances [40].

## Materials and Methods

### Mouse strains.

Genomic rearrangements within the *HoxD* cluster, including deletions (Del) or duplications (Dup), were all produced *in vivo* through targeted meiotic recombination [41], except for the Del(11–13), Del(4–13) and Del(1–13) lines, which were engineered by *loxP/Cre* mediated site-specific recombination in ES cells [42–44]. These three lines were previously referred to as *Del3*, *Del7* and *Del9*, respectively. The Del(10–13), Del(i-9) and Dup(i-9) alleles were previously described [45], whereas Del(12–13), Del(10–11), Del(4–11), and Del(i-13) are novel alleles of the *HoxD* cluster. The Del(12–13), Del(11–13), Del(i-13), Del(4–13) and Del(1–13) alleles also contained the *Hoxd11/lacZ* fusion gene, allowing for histochemical detection of β-galactosidase activity by *X-gal* staining [46]. Targeted insertions of the *Hoxd11/lacZ* and *Hoxd9/lacZ* transgenes to the posterior end of the *HoxD* cluster (TgH[d11/lac] and TgH[d9/lac] strains, respectively) were reported previously [46,47]. The split of the *HoxD* cluster, between *Hoxd11* and *Hoxd10* (Split(10–11), was produced using the sequential targeted recombination-induced genomic (STRING) approach [23].

### Quantitative morphometric analysis.

For histological analyses, newborn kidneys were fixed overnight at 4°C with 4% PFA before alcohol dehydration and paraffin embedding. The whole kidneys were sectioned at 5 μm and stained with hematoxylin and eosin according to standard protocols. Cortical and medullary volumes, as well as glomerular density (number of glomeruli counted per kidney volume) were determined in serial sections throughout entire kidneys according to well established morphometric procedures [48], using the ImageJ free software. For each mouse strain, four kidneys obtained from separate newborn mice were investigated. The independent Student's *t*-test was used for all statistical analyses.

### In situ hybridization.

Embryonic or newborn kidneys were fixed in 4% PFA and cryoprotected in 20% sucrose before embedding in OCT compound. In situ hybridization was performed on 10 μm cryosections by using digoxygenin-labelled riboprobes. Sections were hybridized overnight at 65°C with 2 μg/ml of riboprobe in the following hybridization buffer: 50% formamide, 10% dextran sulfate, 1x Denhardt's solution, 1 mg/ml yeast RNA, 200 mM NaCl, 1.1 mM Tris-base, 8.9 mM Tris-HCl, 5 mM Na_2_HPO_4_, 5 mM NaH_2_PO_4_ and 5 mM EDTA. Two washes of 30 min at 65°C were done in 50% formamide, 1x SSC (0.15 M NaCl plus 0.015 M sodium citrate) and 0.1% Tween 20, followed by two washes at RT in TBS-T (137 mM NaCl, 27 mM KCl, 25 mM Tris-base, 0.1% Tween 20, pH 7.5). Sections were then incubated for one hour in a blocking buffer consisting of 20% goat serum and 2% blocking reagent (Roche) in TBS-T, and then left overnight at 4°C in an alkaline phosphatase-conjugated anti-digoxigenin antibody (Roche) at a dilution of 1:2'000 in the blocking buffer. Five washes of 20 minutes were done in TBS-T, and color development was performed in NTMT (100 mM NaCl, 100 mM Tris, 50 mM MgCl_2_, 0.1% Tween 20, pH 9.5) containing both nitroblue tetrazolium and 5-bromo-4-chloro-3-indolyl phosphate (Roche). The mouse antisense RNA probes for *Hoxd* genes were described previously [45]. The *Gdf5* probe was amplified by PCR, as reported [49].

### Immuno-fluorescence and lectin staining.

Immuno-fluorescent staining on kidney cryosections was carried out as follows: fixation for 10 minutes with 4% PFA; epitope retrieval for 20 minutes at 95°C in 0.01 M citrate buffer (pH 6.0); blockage for one hour with normal goat serum; incubation overnight at 4°C with the primary antibody: anti-CDH1 (1:100, Cell Signaling), anti-ITGα3 (1:400, Chemicon), anti-phospho-SMADs (1:50, Cell Signaling) or anti-C-MYC (1:100, Santa Cruz Biotechnology); incubation with the Alexa Fluor-conjugated secondary antibody (Alexa Fluor-488 or −568, Invitrogen) for one hour. For the detection of GDF5, immuno-fluorescence was performed using a monoclonal anti-GDF5 antibody (20 μg/ml, R&D Systems) and the MOM immuno-detection kit as described by the manufacturer (Vector Laboratories). To assess cell proliferation and apoptosis in kidneys, a double staining was performed with a combination of immuno-staining for anti-phospho-Histone-H3 (Ser10, Upstate) and TUNEL assay (TMR red, Roche) according to the respective manufacturer's instructions. Slides were subsequently counterstained with DAPI (4',6'-diamidino-2-phenylindole) for microscopic observation. Lectin staining was performed using fluorescein isothiocyanate-coupled Dolichos biflorus agglutinin (DBA, Vector Laboratories) as described previously [9].

### Quantitative real-time RT-PCR.

Single-stranded cDNA templates were generated from total kidney RNAs using random hexamers and Superscript II reagents (Invitrogen). For each mouse strain, at least three kidneys from separate embryos or newborn mice were investigated. Quantitative RT-PCR was carried out on an iCycler thermal cycler (Bio-Rad) using SYBR green. PCR efficiencies and dissociation curves of primer pairs were used to ensure specificity of the amplicons. *Tbp*, *Gapdh* and *Tubβ4* genes were used as internal controls for normalization. Primer sequences used for PCR amplification are listed in Table S1. New primer pairs were designed using Primer Express 2.0 software (Applied biosystems).

## Supporting Information

Figure S1 Del(1–13) Mutants Develop Polycystic Kidney Disease Gross appearance of kidneys from control wild-type (WT) and surviving Del(1–13) mutant mice at one month of age. The small size of Del(1–13) kidneys was proportional to the overall reduced body mass of the mutant animals.
(257 KB TIF)

Figure S2 Expression of *Hoxa11/Hoxd11*-Regulated Genes in Del(4–11) and Del(4–13) Mutants The expression pattern of various genes previously shown to be modified in *Hoxa11*/*Hoxd11* double mutant kidneys [17] was assessed by qPCR from E13.5 to P0. Values represent the mean of three independent experiments, after normalization to the WT at day E13.5. Independent Student's *t* test (*HoxD* deletions versus WT): *, P < 0.05.
(955 KB TIF)

Figure S3 Mis-Regulation of *Hoxd13* in the Metanephric Mesenchyme (A) Gross appearance of newborn kidneys from control WT and TgH[d9/lac] heterozygous mice (top panels). The TgH[d9/lac] mouse strain carried a *Hoxd9/lacZ* transgene relocated at the posterior end of the *HoxD* cluster, upstream of the *Evx2* gene [47]. Heterozygous mice were used in this study, because homozygous mutants exhibit complete kidney agenesis at early developmental stages. Kidney sections from WT and TgH[d9/lac] strains were stained with H&E at the newborn stage (middle panels), or were processed for the detection of *Hoxd13* by ISH at E13.5 (*Hoxd13*, bottom panels). The TgH[d9/lac] strain displayed kidney hypoplasia, which coincided with a gain of expression of *Hoxd13* in the mesenchyme.
(B, C) The expression pattern of the indicated genes (from E13.5 to P0) was compared in WT and TgH[d9/lac] heterozygous kidneys by using qPCR. Student's *t* test: *, P < 0.05; **, P < 0.01.
(1.8 MB TIF)

Figure S4 Expression of Renal Developmental Gene Markers in *HoxD* Mutants (A) Expression of various regulators of UB branching was assessed by qPCR from E13.5 to P0. (B) qPCR measurements of the indicated genes were performed in newborn kidneys. Independent Student's *t* test (*HoxD* deletions versus WT): *, P < 0.05; **, P < 0.01.
(1.0 MB TIF)

Figure S5 Posterior *Hoxd* Genes Are Not Affected in Del(i-9) Mutant Kidneys Kidney cryosections of WT and Del(i-9) homozygous strains were processed for the detection of *Hoxd10* by ISH at E13.5 (top panels), and qPCR was used to compare the expression levels of *Hoxd12*, *Hoxd11* and *Hoxd10* (bottom panels). Student's *t* test: *, P < 0.05.
(971 KB TIF)

Table S1 Primers for Real-Time RT-PCR Sequence of primers used in quantitative real-time RT-PCR. Primer pairs described previously are indicated in the last column.
(28 KB XLS)

## Abbreviations

CDH1: cadherin type 1
DBA: *Dolichos bifluoris* agglutinin
E: embryonic day
GBM: glomerular basement membrane
GDF: growth and differentiation factor
H&E: hematoxylin and eosin
ISH: *in situ* hybridization
ITGα3: integrin-α3
MM: metanephric mesenchyme
P: postnatal day
P-H3: phosphorylated histone H3
PKD: polycystic kidney disease
P-SMAD: phosphorylated SMAD
qPCR: quantitative RT-PCR
UB: ureteric bud
WT: wild-type
